# Writing for Health: Rationale and Protocol for a Randomized Controlled Trial of Internet-Based Benefit-Finding Writing for Adults With Type 1 or Type 2 Diabetes

**DOI:** 10.2196/resprot.7151

**Published:** 2017-03-14

**Authors:** Joanna Crawford, Kay Wilhelm, Lisa Robins, Judy Proudfoot

**Affiliations:** ^1^ Faces in the Street Urban Mental Health Research Institute St. Vincent's Health Australia Sydney Australia; ^2^ School of Psychiatry Faculty of Medicine University of New South Wales Sydney Australia; ^3^ Consultation Liaison Psychiatry St. Vincent's Health Australia Sydney Australia; ^4^ Black Dog Institute Sydney Australia

**Keywords:** type 1 diabetes, type 2 diabetes, diabetes-related distress, writing, Internet intervention, randomized controlled trial

## Abstract

**Background:**

Diabetes mellitus is Australia’s fastest growing chronic disease, and has high comorbidity with depression. Both subthreshold depression and diabetes distress are common amongst people with type 1 or type 2 diabetes, and are associated with poorer diabetes self-care. A need exists for low-intensity self-help interventions for large numbers of people with diabetes and diabetes distress or subthreshold depression, as part of a stepped-care approach to meeting the psychological needs of people with diabetes. Benefit-finding writing is a very brief intervention that involves writing about any positive thoughts and feelings about a stressful experience, such as an illness. Benefit-finding writing has been associated with increases in positive affect and positive growth, and has demonstrated promising results in trials amongst other clinical populations. However, benefit-finding writing has not yet been examined in people with diabetes.

**Objective:**

The aim of this randomized controlled trial (RCT) is to evaluate the efficacy of an Internet-based benefit-finding writing (iBFW) intervention for adults with type 1 or type 2 diabetes (compared to a control writing condition) for reducing diabetes distress and increasing benefit-finding in diabetes, and also improving a range of secondary outcomes.

**Methods:**

A two-arm RCT will be conducted, using the online program Writing for Health. Adults with type 1 or type 2 diabetes living in Australia will be recruited using diabetes-related publications and websites, and through advertisements in diabetes services and general practitioners’ offices. Potential participants will be referred to the study-specific website for participant information and screening. All data will be collected online. Participants will be randomized to either iBFW about diabetes, or a control writing condition of writing about use-of-time. Both conditions involve three daily sessions (once per day for three consecutive days) of 15-minute online writing exercises. Outcome measures will be administered online at baseline, one-month, and three-month follow-ups.

**Results:**

This trial is currently underway. The primary outcomes will be diabetes distress and benefit-finding in diabetes. Secondary outcomes will be depression, anxiety, diabetes self-care, perceived health, and health care utilization. We aim to recruit 104 participants. All stages of the study will be conducted online using the Writing for Health program. Group differences will be analyzed on an intention-to-treat basis using mixed models repeated measures. Linguistic analyses of the writing exercise scripts, and examinations of the immediate emotional responses to the writing exercises, will also be undertaken.

**Conclusions:**

This RCT will be the first study to examine iBFW for adults with type 1 or type 2 diabetes. If iBFW is found to be efficacious in reducing diabetes distress and improving diabetes self-care and other outcomes, iBFW may offer the potential to be a low-cost, easily accessible self-help intervention to improve the wellbeing of adults with diabetes.

**Trial Registration:**

Australia and New Zealand Clinical Trials Registry (ACTRN12615000241538)

## Introduction

### Background

#### Need for Cost-Effective Psychological Interventions for People with Diabetes

Diabetes mellitus is a global public health challenge. Diabetes is a leading cause of disease burden worldwide [1] and is increasing in prevalence, with an estimated 422 million adults having diabetes in 2014 [2]. The cooccurrence of diabetes and depression is common, with the prevalence rates of depression and anxiety at least twice as high in patients with type 1 or type 2 diabetes compared to the general population worldwide [3-5]. Depression is associated with poor diabetes self-management [6,7] and increased disease severity, complications, and mortality [8,9]. Subthreshold depression (clinically significant symptoms of depression that do not meet diagnostic criteria for a major depressive episode or dysthymia) is more common in people with diabetes than major depression, with approximately half of all adults with type 2 diabetes experiencing at least one episode of subthreshold depression over five years [9]. Even subthreshold depression in diabetes is associated with poorer quality of life [10] and reduced adherence to diabetes self-care (including exercise, diet, and medication) [11], in addition to being a risk factor for future major depression [12].

Diabetes distress is a construct partly overlapping with depression in people with diabetes, and includes negative thoughts and emotions towards diabetes and its treatment [13]. Approximately 10-30% of people with diabetes experience severe diabetes distress [14,15], yet many of these people are not clinically depressed. Approximately 70% of people with type 2 diabetes display high levels of diabetes-related distress without meeting criteria for major depressive disorders [16,17]. Diabetes distress is associated with poor glycemic control, acting as a unique contributor to poor self-care adherence [18]. Diabetes distress is also a risk factor for the incidence and persistence of depressive symptoms [19].

Thus, international guidelines for diabetes management now recognize the importance of psychological care, not only to improve quality of life, but also diabetes self-management and medical outcomes [20]. Screening for both depression and diabetes distress, followed by appropriate interventions, has been recommended [21,22]. A stepped-care approach to the management of depression in people with diabetes has been suggested, with mild or subthreshold symptoms of depression managed within primary care, utilizing evidence-based self-help interventions [23]. This approach is in line with recommendations by the UK National Health Service, and Diabetes UK, for low-intensity psychological interventions to be used for people with diabetes with lower-level depression or distress [24].

Given the large numbers of people affected by diabetes globally, accessibility and cost-effectiveness are key issues in their psychological care. The Internet is an increasingly popular and cost-effective method of increasing access to evidence-based psychological interventions, and overcomes several of the traditional barriers to accessing mental health care, such as cost and concerns about stigma and privacy [25]. The Internet offers great potential for public health and prevention interventions [25]. For people with diabetes, Internet-based programs have demonstrated user acceptability and potential efficacy for improving diabetes self-management [26,27], and efficacy in reducing depression, anxiety, and diabetes distress [28-30]. Therefore, brief, Internet-based interventions have the potential to offer low-cost assistance to large numbers of people with diabetes who are experiencing mild or subthreshold psychological symptoms, as part of a stepped-care approach.

#### Evolution of Expressive Writing as a Brief Intervention

Therapeutic writing is a brief intervention that aims to improve physical or mental health [31]. The most common form of therapeutic writing is expressive writing (EW), in which thoughts and feelings regarding a stressful event are disclosed in writing, typically for 15-20 minutes for three to four days within a short period of time [32]. EW has been examined in over 250 studies investigating its effects on physical and/or mental health in a wide range of populations, including healthy participants, people with psychological problems, or people with long-term health conditions such as chronic pain, asthma, cancer, cystic fibrosis, or arthritis [31,33]. Mental health benefits of EW have included reduced symptoms of depression [34,35], anxiety [36], and posttraumatic stress [37,38]. Physical health benefits of EW have included improved lung function in asthma patients [39], improved immune function in patients with human immunodeficiency virus infection [40], and reduced fatigue and pain in adults with lupus or rheumatoid arthritis [41]. Evidence for behavioral change following EW also exists, such as decreased health care utilization [32,42], reduced aggression in adolescents [43], and improved exam performance [44]. Several reviews and meta-analyses of EW studies are available [31,33,39,45,46].

However, there are limitations to EW. Results of EW studies are quite variable, and effect sizes are often small. Meta-analyses of the effects of EW have found overall small effects of EW for distress (*r*=.102) [33] and physical health in medically ill populations (Cohen *d*=.21) [45]. The mechanisms of EW remain unclear [31], and tend to differ depending on the exact instructions that are used [47]. Furthermore, EW often involves an immediate increase in negative mood [31], even when followed by longer-term psychological benefits [46]. Thus, it has been suggested that EW in vulnerable populations is best undertaken with therapist support and follow-up [46].

Indeed, pilot trials of EW in people with diabetes have yielded mixed results. A pilot trial of 22 participants with type 1 diabetes randomized to either an EW group (instructed to write about an emotional or stressful topic *related to diabetes* for 20 minutes each day over four consecutive days) or a control group (instructed to write about factual topics related to diabetes) found that, at three-months follow-up, the EW group experienced less depressive symptoms and fewer incidences of physical illness [48]. The difference between the two groups in mean self-recorded blood glucose levels (effect size *r*=.236) was in the direction of benefit to the EW group, although this difference was not statistically significant [33]. However, a pilot trial of 41 adults with type 2 diabetes randomized to either EW or neutral writing found that EW was associated with a *worsening* in depressive symptoms, with no change in diabetes distress [49]. Of note, in the latter study the EW task involved writing about any stressful experience over the past month rather than a diabetes-specific task.

These findings have led researchers to investigate other variations of therapeutic writing, to maximize benefits and increase positive affect (and reduce distress) during the intervention. By modifying writing instructions, researchers can attempt to increase the likelihood that participants engage in desired cognitive processes, and thereby aim to increase the benefits gained from the writing task [38,47].

#### Benefit-Finding Writing

Benefit-finding writing involves participants writing about any *positive* thoughts and feelings about a stressful experience, such as an illness. Until recently, research has largely overlooked the utility of positively-focused writing following stressful events or illness [50]. However, there is emerging evidence that the experience of a medical illness often has sequelae that patients view as positive or beneficial [51]. Increased recognition has been given to the concept of *benefit-finding*, defined as, “identifying positive life changes resulting from adversity and negative life stressors, including illness” [52]. Correlated with posttraumatic growth (positive changes in individuals following traumatic life events) [53], benefit-finding has been associated with increased psychosocial wellbeing and decreased depression in a range of clinical populations [52,54], including people with diabetes [55]. Benefit-finding has also been linked with increased optimism, positive affect [56], self-efficacy, and adaptive coping strategies [57]. Benefit-finding in diabetes has been associated with lower symptoms of depression, increased adherence to diabetes self-care, and greater perceived coping effectiveness [55]. Furthermore, benefit-finding amongst parents of children with diabetes has also been associated with better glycemic control in their children [58]. It has therefore been suggested that interventions could be developed to increase benefit-finding in people with diabetes [55].

Benefit-finding writing aims to improve other outcomes by facilitating increased ongoing benefit-finding in relation to a medical condition or stressor. However, this concept has not yet been examined in people with diabetes. To date, benefit-finding writing has been examined in student and community samples [47,59-61], bereaved undergraduate students [62], participants who had recently experienced a relationship dissolution [50], breast cancer patients [42], and adults with lupus or rheumatoid arthritis [41].

Trials in nonclinical populations have found that benefit-finding writing results in less distress and increased positive affect immediately postwriting, compared to EW [60,61]. Benefit-finding writing is associated with greater increases in posttraumatic growth [47], and greater use of cognitive-insight words, compared to standard EW [61].

Two trials in clinical populations have compared benefit-finding writing with EW, with promising results [41,42]. In women with early-stage breast cancer, both benefit-finding and expressive-writing groups had significantly fewer medical appointments for cancer-related morbidities, relative to the control group [42]. In adults with lupus or rheumatoid arthritis, both benefit-finding writing and EW groups had lower fatigue at three months, relative to a control writing group [41]. An interaction with trait anxiety was also found; benefit-finding appeared to be more useful in reducing pain for those with high trait anxiety [41]. Furthermore, the authors noted that all 27 participants in the benefit-finding group were able to write some, “positive thoughts and feelings” about their illness experience [41]. Thus, the limited research on benefit-finding writing to date suggests that it may have the same longer-term health benefits as EW, but with the added advantage of immediate increases in *positive* affect

#### Rationale for Current Study

EW is a brief, low-cost intervention that can be delivered via the Internet [63-65]. These factors potentially make EW suitable as a short-term, low-intensity intervention to supplement treatment-as-usual for people with diabetes who have lower-level psychological needs [49]. While the results of pilot trials of EW in diabetes are mixed [48,49], benefit-finding writing is a more recent variation of therapeutic writing, which aims to facilitate increased perceptions of positive life changes resulting from adversity and negative life stressors. As outlined above, the limited research on benefit-finding writing suggests that it leads to increases in positive affect and posttraumatic growth, and may have the same physical health benefits as EW in populations with medical conditions. However, benefit-finding writing has not yet been examined in people with diabetes (type 1 or type 2).

Therefore, the current study aims to examine the feasibility and efficacy of an Internet-based benefit-finding writing (iBFW) intervention for adults with type 1 or type 2 diabetes. We seek to evaluate the efficacy of iBFW (compared to a control writing condition) in reducing diabetes distress and increasing benefit-finding in diabetes, and also in reducing symptoms of depression and anxiety, improving diabetes self-care and self-rated health, and improving health-care utilization. This paper presents the study protocol for this randomized controlled trial (RCT), using the online program *Writing for Health*.

### Study Aims and Hypotheses

The primary outcomes of this RCT will be the impact of the iBFW, compared to a control writing condition, on diabetes distress and benefit-finding in diabetes. Our primary hypotheses are that adults with type 1 or type 2 diabetes randomized to receive iBFW will demonstrate significantly reduced diabetes distress and significantly increased benefit-finding for diabetes, compared to the control condition, at both one-month and three-month follow-ups.

Secondary outcomes will include symptoms of depression, symptoms of anxiety, diabetes self-care, health care utilization, and perceived self-health. Our secondary hypotheses are that compared to those in the control condition, the iBFW group will demonstrate significant: (1) reductions in depression symptoms; (2) reductions in anxiety symptoms; (3) increased diabetes self-care; (4) reduced number of visits to health professionals; and (5) improved perceived health, at both one-month and three-month follow-ups.

This study also aims to examine validation of the intervention instructions by investigating immediate emotional responses to the writing tasks, and the number of positive emotion words and cognitive insight words used in the writing tasks. It is hypothesized that compared to those in the control condition, the iBFW group will (1) show greater increases in positive affect postwriting, and (2) use more positive emotion words and more cognitive insight words than the control group.

## Methods

### Study Design

**Figure 1:**
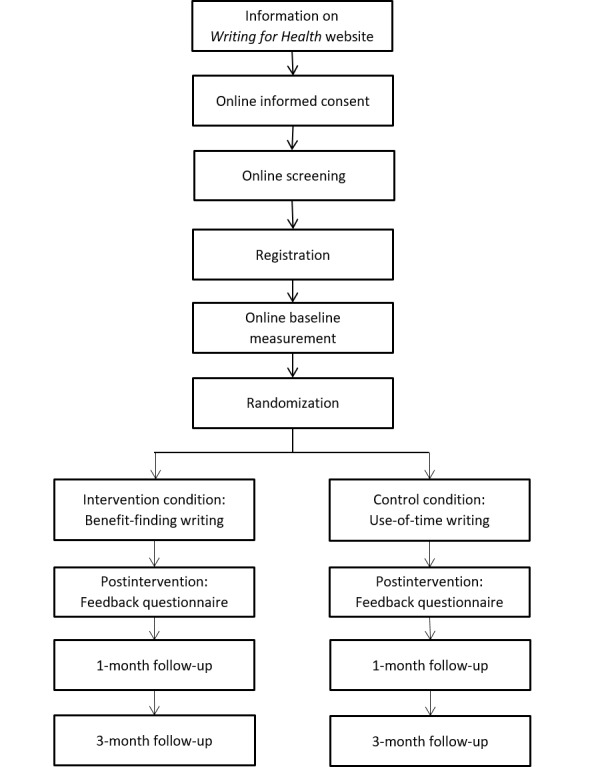
Study flow chart.

A 2 (conditions) x 3 (time) RCT design is planned. A flow diagram for the trial is shown in Figure 1. Participants will be randomized to either iBFW or an Internet-based control writing condition. Both conditions involve an intervention of 3 days of online writing. Outcomes will be assessed at 3 time points for both groups: baseline, one-month, and three-months postintervention. We will also assess self-rated current mood immediately prior to and following each writing session, and administer three survey questions (assessing how personal, meaningful, and distressing the writing exercise was) after each writing task. An online *Feedback Questionnaire* to assess user satisfaction and perceived helpfulness will also be administered postintervention.

**Figure 2:**
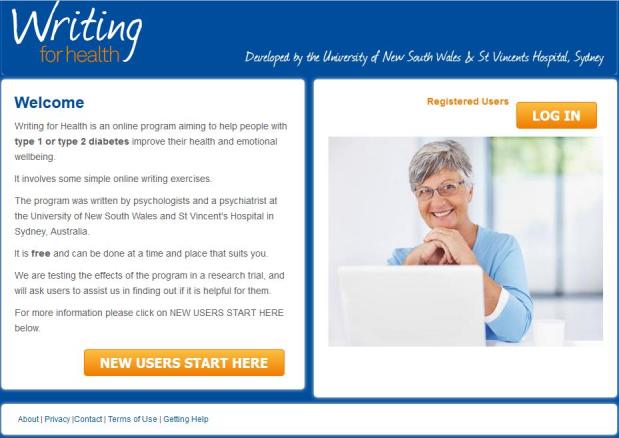
Screenshot of the homepage of Writing for Health.

### Participants

#### Recruitment

Participants will be recruited online from Australian diabetes-related websites and publications, and from advertisements in waiting rooms of diabetes services and general practitioners (GPs) throughout Australia. Participants will apply for the study via the *Writing for Health* website [66], where they will complete an automated screening questionnaire (which also provides baseline data) after reading the study information and provide informed consent. Excluded applicants will immediately receive an onscreen message that informs them that the program is not suitable for them, and will provide links to appropriate resources. All potential participants will be provided with feedback on the severity of their depression and anxiety symptoms. Participants who meet eligibility criteria will proceed to online registration with the program, complete further online questionnaires (for further baseline data), be automatically randomized, and then complete the first writing session.

#### Eligibility

The inclusion and exclusion criteria and summarized in Textbox 1. Screening will be conducted online in the *Writing for Health* program. If any responses indicate ineligibility, screening will be automatically stopped and the next onscreen page will provide appropriate feedback (including links to relevant resources).

**Inclusion and exclusion criteria.**

Inclusion criteria:

- Consent to participate
- Aged 18 years or older
- Living in Australia
- Type 1 or type 2 diabetes, diagnosed by a general practitioner or endocrinologist
- Email address and access to the Internet

Exclusion criteria:

- Inability to read or write in English with ease
- Patient Health Questionnaire-9 (PHQ-9) score >10 and/or Generalized Anxiety Disorder-7 (GAD-7) score >8
- Diagnosis of bipolar disorder or a psychotic disorder
- Diagnosis of dementia or another cognitive disorder
- Current psychological therapy

#### Ethics

This study protocol has been approved by the Ethics Committee at St. Vincent’s Hospital, Sydney, which is certified by the National Health and Medical Research Council in Australia (HREC/13/SVH/379). This trial was prospectively registered with the Australia and New Zealand Clinical Trials Registry (ACTRN12615000241538).

### Intervention and Control

#### Writing for Health

This RCT will be conducted using the online program *Writing for Health* [66], which was developed for this study by mental health researchers (including psychologists and a psychiatrist) at St. Vincent’s Hospital, Sydney, and the University of New South Wales. All stages of this study will be conducted online through *Writing for Health*, including information about the study, consent to participate, screening questionnaires with automated feedback, participant registration, randomization to one of two conditions, the writing intervention, and follow-up questionnaires. Automated reminder emails will be sent by *Writing for Health* to participants on each day of the intervention, and when it is time to complete their follow-up questionnaires.

*Writing for Health* provides minimal clinician assistance. Direct contact between participants and the clinicians will not occur in the standard course of the trial. Psychologists will monitor participant responses and distress levels throughout the intervention, and in accordance with the risk management protocol, will correspond with participants by email and phone to assess any support needed, and refer to appropriate services if required.

Participants registered with *Writing for Health* will be randomized to one of two conditions: iBFW or Internet-based use-of-time writing (control condition). Participants from both conditions will continue to receive usual care from their health services. Following randomization, both conditions involve participants writing online (in the *Writing for Health* program) for 15 minutes once a day, according to instructions provided. There will be three daily 15-minute online writing sessions (once per day for three consecutive days.) A timer on the screen counts down from 15 minutes, to allow participants to keep track of time during their writing session.

Information provided to participants in *Writing for Health* describes the aim of this study as investigating whether the writing exercises in the *Writing for Health* program improve the mental and physical wellbeing of people with diabetes. Participants will be informed that they will be randomized to one of two types of writing exercises, and both types of writing exercises will be described. However, research hypotheses will not be revealed.

#### Intervention Condition - Internet-Based Benefit-Finding Writing for Diabetes

Participants in the iBFW condition will be asked to write about any *positive* thoughts and feelings that they have had about their experiences with diabetes. The instructions (see Multimedia Appendix 1) are adapted from those used by Stanton and colleagues (2002) in benefit-finding writing for women with breast cancer [42]. The same instructions will be provided for all three of the writing sessions, consistent with previous studies of benefit-finding writing [41,42].

#### Control Condition - Internet-Based Use-of-Time Writing

Participants in the control condition will be asked to write in detail about how their time was spent that day (first writing session) and plans for how their time will be spent the following day (second writing session) and week (third writing session). Participants will be instructed to be as objective as possible, and to focus on the facts and details of how their time was spent (or will be spent), and not to focus on their emotions or opinions. Writing about use-of-time is a neutral topic that has been previously used as an active control condition in a trial of EW, and was found to be associated with reduced physical and mental health symptoms [63].

### Procedure

Potential participants will visit the *Writing for Health* website [66], read the participant information, and indicate their consent to participate by checking a box. Potential participants will complete screening questionnaires with automated feedback, and if eligible, can then register to participate. Following completion of further online questionnaires, participants will be automatically randomized to one of two conditions: iBFW or control writing. Participants can then proceed to the first session of their 3-day writing intervention (either iBFW or control writing). Immediately before and after each of their three 15-minute writing sessions, participants will be asked to rate their current mood. In addition, following each writing session, participants will be asked to rate how personal, meaningful, and distressing their writing session was that day, and can comment on the writing session if they wish. At the completion of their three-day writing intervention, participants will be asked to complete the online *Feedback Questionnaire*, to assess user satisfaction and perceived helpfulness. Outcome measures will be administered in the online *One-Month* and *Three-Month Follow-Up Questionnaires* (in addition to baseline).

Participants will be sent automatic reminder emails by *Writing for Health* on day 2 and day 3 of their writing intervention, and also one-month and three-months postintervention, prompting participants to complete their follow-up questionnaires. All participants will be provided with automatic feedback of the range in which they scored on depression and anxiety measures, at baseline, one-month, and three-month follow-ups.

### Risk Management Protocol

Psychologists will monitor participant responses and distress levels throughout the intervention. Although direct contact with participants will not occur during the standard course of the trial, a psychologist or psychiatrist will contact participants by email and/or telephone in certain circumstances, as outlined below. After each writing session, participants will be required to rate how distressing the writing session was on a 6-point scale. If participants indicate that a writing session was at all distressing (by responding 1 or greater), then the *Writing for Health* program will automatically display a feedback page outlining strategies to manage distress and suggest that they contact their GP if further support is needed. This page also includes the telephone number of a 24-hour telephone mental health helpline available in Australia (Lifeline). If the participant responds with a distress rating of 5 or 6 after any writing session, they will also be emailed by a psychologist, with further telephone contact based on the clinical discretion of study psychologists and psychiatrist.

Participants’ depression and anxiety scores on the Patient Health Questionnaire-9 (PHQ-9) and Generalized Anxiety Disorder-7 (GAD-7), respectively, will also be monitored. If a participant’s score is in the severe range for depression or anxiety at one-month or three-month follow-up and/or the participant indicates possible suicidal thoughts by responding 1 or greater on item 9 of the PHQ-9 (“Over the past two weeks, have you been bothered by… thoughts that you would be better off dead, or of hurting yourself in some way?”), the participant will be emailed and then telephoned by a *Writing for Health* psychologist to assess the supports the participant is receiving, and provide contact details or referrals to appropriate services. In addition, the *Writing for Health* program will provide automatic feedback pages at the end of the follow-up questionnaire sessions for participants who have scored in the above ranges, providing information on how to access mental health support, including the recommendation to contact their GP and the telephone number for Lifeline. Similarly, at the final three-month follow-up, the same procedure will apply to participants who score in the moderate or greater range for depression or anxiety on the PHQ-9 or GAD-7, respectively.

### Randomization

Randomization to the two groups will be automatically generated by the *Writing for Health* content management system after participants have registered with the program. Randomization is therefore concealed to the researchers.

### Primary Outcome Measures

#### Diabetes Distress

The Diabetes Distress Scale (DDS17) [67] is a 17-item self-report measure of psychosocial stress associated with diabetes, with four reliable subscales: emotional burden (feeling overwhelmed by diabetes), physician-related distress (worries about access, trust, care), regime-related distress (concerns about diet, physical activity, medications), and interpersonal distress (not receiving understanding and appropriate support from others). Cut-off points on the DDS17 have been established for little or no distress, moderate distress, and high distress [68].

#### Benefit-Finding

The 17-item Benefit Finding Scale [51] was developed to investigate benefit-finding in women with early stage breast cancer. In the current study, the stem question is modified from, “Having had breast cancer has…” to, “Having had diabetes has…” Participants are asked to respond to each of the 17 perceived benefits, such as, “has lead me to be more accepting of things” and, “has brought my family closer together” on a five-point scale with labels of *not at all* (1), *a little* (2), *moderately* (3), *quite a bit* (4), and *extremely* (5). This scale has previously been adapted for use in diabetes (with one item removed) and found to have one large factor and good internal consistency (Cronbach alpha=.89) in a population of adolescents with type 1 diabetes [55]. Table 1 provides an overview of all measurement tools and administration time-points.

**Table 1:** Measurement tools and questions at each time-point.

		Questionnaires	Baseline	Pre-writing session	Post-writing session	Post-final session	1-month follow-up	3-month follow-up
**Demographics**			✓					
**Primary outcomes**								
	Diabetes distress	Diabetes Distress Scale	✓				✓	✓
	Benefit finding	Benefit Finding Scale	✓				✓	✓
**Secondary outcomes**								
	Depression	Patient Health Questionnaire-9	✓				✓	✓
	Anxiety	Generalized Anxiety Disorder-7	✓				✓	✓
	Diabetes self-care	Summary of Diabetes Self-Care Activities Measure (Revised)	✓				✓	✓
	Self-rated health	Single item	✓				✓	✓
	Health care utilization	Single item	✓				✓	✓
	Positive and negative affect	International Positive and Negative Affect Schedule Short Form		✓	✓			
	Experiences during writing session	Questions assessing how personal, meaningful, and distressing the writing session was			✓			
	User satisfaction	Feedback Questionnaire				✓		

### Secondary Outcome Measures

#### Depression Symptoms

The PHQ-9 [69] is a brief, widely used, reliable, and valid 9-item self-report that measures both the severity of depression over the preceding two weeks and diagnosis of depression based on criteria of the Diagnostic and Statistical Manual of Mental Disorders, 4^th^Edition (DSM-IV). This questionnaire has established cut-off scores of 5, 10, 15, and 20, representing *mild*, *moderate*, *moderately severe*, and *severe* depression. The total score ranges between 0 and 27, with scores equal or above 10 having a sensitivity of 88% and a specificity of 88% for major depression [69].

#### Anxiety Symptoms

The GAD-7 [70] is a brief, widely used, reliable, and valid 7-item self-report that measures the severity of anxiety. Scores on the GAD-7 range from 0 to 21; scores of 5, 10, and 15 represent *mild*, *moderate,* and *severe* anxiety symptoms. A total score of 8 on the GAD-7 has been identified as an important threshold for identifying the presence of an anxiety disorder [71].

#### Positive and Negative Affect

The International Positive and Negative Affect Schedule Short Form (I-PANAS-SF) [72] is a reliable and valid 10-item measure of positive and negative affect, which is comprised of 10 words that represent positive and negative affect. The correlations that this scale has with the positive and negative affect scales of the full 20-item form of the Positive and Negative Affect Schedule (PANAS) are .92 and .95, respectively [72]. Instructions were modified to assess state rather than trait affect, using the instructions of the 20-item PANAS-Immediate Version [73]. Participants will be instructed to indicate the degree of specific affect they feel, “right now, at the present moment”, on a scale of 1 to 5 (1=very slightly/not at all; 5=extremely).

#### Diabetes Self-Care

The Summary of Diabetes Self-Care Activities Measure (Revised) [74] is an 11-item self-report measure of self-care of diabetes mellitus (including diet, exercise, blood sugar testing, foot care, and smoking) that is widely used both clinically and in research. Items in the revised version were selected based on their psychometric properties, sensitivity to change, and ease of scoring and interpretation [74]. In a critical appraisal of 26 different measures of diabetes outcomes, the Summary of Diabetes Self-Care Activities Measure (Revised) was one of only three measures to meet all criteria of suitability, validity, reliability, and sensitivity to change [75].

#### Self-Rated Health

Self-rated health will be assessed by the question, “In general, how would you rate your health at present?” The five response options are *very good*, *good*, *fair*, *poor,* and *very poor*. Responses to this question have previously been found to be significantly associated with blood glucose indicator hemoglobin A1c (HbA1c; with poorer self-rated health associated with higher HbA1c levels) and number of self-reported diabetes-related symptoms in patients with type 2 diabetes [76].

#### Health Care Utilization

Participants will be asked to answer the question, “In the past month, how many times have you visited a doctor or other health care professional?” This same question will be administered at three time-points: baseline, one-month follow-up, and three-month follow-up.

### Additional Measurements

We will collect sociodemographic information (age, gender, education, and occupation), diabetes-related information (type, duration of illness, management, and complications), and participant feedback about the program.

#### Experiences During Writing Session

Immediately after each writing session, participants will be asked to rate how meaningful, personal, and distressing their writing exercise was, on a 7-point scale (0=not at all; 6=extremely). Similar questions have been used as manipulation checks in previous studies of therapeutic writing [42,77]. In addition, participants in the iBFW intervention condition will be asked immediately after each writing session if they were able to identify *any* positive thoughts or feelings about living with diabetes in their writing session.

#### Feedback Questionnaire

A 12-item self-report questionnaire was developed to assess participants’ experiences and perceptions of the *Writing for Health* program. Item content was informed by self-report measures from other evaluations of Internet-based interventions. Items 1 to 6 ask participants to rate responses on a 5-point scale (from *not at all* to *very*) regarding aspects of usability and perceived helpfulness of the *Writing for Health* program, including how easy to use it was. Items 1 to 3 are taken from the Internet Intervention Evaluation Questionnaire [78]. Items 4 and 5 are modified from items previously used to assess credibility of writing interventions (asking participants to rate how logical the writing exercises seemed and how confident they would be in recommending it to a friend) [79]. Item 6 examined the perceived helpfulness of the writing exercises in reducing stress. Item 7 examined technical difficulties with the online program, and items 8-12 were open-ended questions examining the most helpful and least helpful aspects of the program, and any suggested improvements.

## Results

### Sample Size

EW studies have typically had modest effect sizes, with meta-analyses reporting small effects of EW for distress (*r*=.102) [33], and for physical health in medically ill populations (Cohen *d*=.21) [45]. However, of the few benefit-finding writing studies published, effect sizes appear to be greater than those for EW. Benefit-finding writing has been found to have large within-group effect sizes (Cohen *d=*.64-1.22) and small-to-moderately large between-group effect sizes (Cohen *d*=.20-0.66) for improving symptoms of complicated grief, posttraumatic stress disorder, and physical health in bereaved adults (calculated based on means and standard deviations reported in Lichtenthal et al 2010) [62]. Such interventions have also been found to have large between-group effect sizes (Cohen *d*=.68-2.4) for improvements in somatic symptoms and reduction in medical appointments in women with breast cancer (calculated based on means and standard deviations reported in Stanton et al 2002) [42].

A recent review of therapeutic writing called for future studies to conduct feasibility or pilot studies in new clinical populations, prior to full evaluations with sufficient statistical power to detect modest effect sizes [31]. Given that no previous studies have examined benefit-finding writing in people with diabetes, it would be prudent to first conduct a pilot RCT to examine its feasibility and a preliminary investigation of its efficacy. Other pilot trials of therapeutic writing in clinical populations have taken a similar approach [64,65,80]. Thus, a very large sample required to detect a small effect size is beyond the scope of this initial study.

Given that this study is partially exploratory, we therefore decided to recruit a sample size with sufficient power to detect a moderately large between-groups effect size (Cohen *d*=.7). Based on statistical power of 0.8 and probability level of *P*<.05, a sample size of 26 per group (that is, 52 for each of the two groups) will be needed for one-tailed tests. Given the expected attrition rate of up to 50% [81], our target total sample size is therefore 104 individuals.

### Statistical Analyses

Statistical analyses will be conducted using SPSS 22 software. Group differences in demographic data, diabetes-related variables, and baseline measures will be analyzed using one-way analysis of variance (continuous variables) and chi-square tests (categorical variables). Similar analyses will be conducted to compare participants who do (nondropouts) and do not (dropouts) complete all questionnaires at each of the time-points, to explore possible biases in study attrition. Analyses will be conducted to validate the writing intervention instructions in several ways:

To examine immediate emotional responses to the writing interventions, scores on the I-PANAS-SF [72] administered immediately before and after each writing session will be analyzed using a 2 (group) x 3 (session) x 2 (positive affect and negative affect) repeated measures multivariate analysis of variance. This test will be used to investigate the hypothesis that the benefit-finding group will have greater increases in positive affect postwriting, relative to the control group.

The content of the written scripts in both groups will be assessed using the Linguistic Inquiry Word Count 2007 software program [82], to examine differences in positive emotion words and cognitive insight words. This validated method provides a content analysis of the language used in the scripts, and quantifies the number of words used from specific categories (eg, emotions, cognitive processes). This approach will be used to investigate the hypothesis that the benefit-finding group will use more positive emotion words and more cognitive insight words than the control group.

Scores on the feedback questionnaire, a measure developed to assess user satisfaction and perceived usefulness of the intervention, will be compared between the two groups using analyses of variances. These tests will be used to examine the hypothesis that the participants in the benefit-finding group will have higher levels of user satisfaction and perceived helpfulness of the writing tasks, relative to the control group.

Outcome data at the one-month and three-month follow-up time-points will be analyzed on an intention-to-treat basis using linear mixed modelling, with time-points as a within-group factor and intervention as a between-group factor. The interaction of time and study condition will be examined in each analysis, as a significant interaction will indicate a group difference in the pattern of change over time in the outcome of interest. Significant interactions will be explored using Bonferroni adjusted comparisons of the two groups at one-month and three-month follow-ups. All effects will be tested at *P*<.05. Within-group and between-group Cohen *d* effect sizes will be calculated.

### Trial Status

The trial is currently in the data collection phase. Recruitment to the study commenced in February 2015. Results are expected by July 2017.

## Discussion

This study will be the first to examine benefit-finding writing for adults with type 1 or type 2 diabetes. The feasibility and efficacy of this brief intervention will be evaluated in a two-arm RCT, with a three-month follow-up period, in which iBFW for diabetes is compared to an active control condition (use-of-time writing). The participants in this study will be adults with type 1 or type 2 diabetes who may be experiencing diabetes distress and/or mild symptoms of depression or anxiety. Participants with both type 1 and type 2 diabetes will be included in this study, as perceived benefits of living with diabetes have previously been reported by both people with type 1 diabetes [55] and type 2 diabetes [83]. Outcomes assessed will include multiple psychological and diabetes-specific variables, including the primary outcomes of diabetes distress and benefit-finding for diabetes, and secondary outcomes of symptoms of depression and anxiety, diabetes self-care, perceived health, and health care utilization. Furthermore, we will investigate validation of the intervention by examining immediate emotional responses to the writing tasks and conduct linguistic analyses of the writing scripts.

Results from this trial will contribute to the growing body of knowledge about a more recent form of therapeutic writing, known as benefit-finding writing. The limited research on benefit-finding writing to date suggests that it may have the same health benefits of the more commonly researched EW, but with the advantage of increased positive affect immediately following the intervention.

Limitations to this study include the brevity of the follow-up period (three months) and the reliance on self-reported data. Physiological data, such as HbA1c or other indicators of blood glucose level, are not included in this study. The included outcome of health utilization has limitations itself, as participants may not visit health professionals frequently enough for any changes to be detected in the follow-up period of three months. Furthermore, it is unclear as to whether decreased health care utilization is a positive outcome, given that going to an appropriate health professional when a need exists is a good thing [84]. Nevertheless, we have included assessment of this outcome as any changes in health care utilization in people with diabetes would be of interest, and previous trials of EW and benefit-finding writing have reported reductions in health care utilization [42,84].

Similar to many RCTs, the generalizability of our results is restricted by the exclusion criteria. For example, adults with diabetes currently experiencing depression of moderate or greater severity will be excluded from this trial; hence the results will not be able to be generalized to people with diabetes who are currently depressed. Furthermore, the sample size (N=104) will enable the detection of a moderately large effect size, in line with some previous studies of benefit-finding writing [42], but will not allow for the detection of small effect sizes reported in meta-analyses of writing interventions. However, as recommended in a recent comprehensive review of therapeutic writing [31], when investigating new writing interventions in new clinical populations, it is prudent to first conduct feasibility studies and pilot trials. Thus, this novel study will enable a preliminary investigation of the feasibility and efficacy of benefit-finding writing for adults with diabetes.

If the iBFW is found to be helpful for people with type 1 or type 2 diabetes, this intervention will offer the potential to be a low-cost, easily accessible public health intervention to improve the well-being of large numbers of diabetic patients with lower-level psychological needs. Furthermore, benefit-finding writing may also have the potential to assist other populations with chronic conditions.

## Appendix

Multimedia Appendix 1 Instructions for Internet-based benefit-finding writing (iBFW) for diabetes.

## Abbreviations

DDS17: Diabetes Distress Scale
EW: expressive writing
GAD-7: Generalized Anxiety Disorder-7
HbA1c: hemoglobin A1c
iBFW: Internet-based benefit-finding writing
I-PANAS-SF: International Positive and Negative Affect Schedule Short Form
PANAS: Positive and Negative Affect Schedule
PHQ-9: Patient Health Questionnaire-9
RCT: randomized controlled trial
